# Catalytic pyrolysis (Ni/Al-MCM-41) of palm (*Elaeis guineensis*) oil to obtain renewable hydrocarbons

**DOI:** 10.1039/d0ra06122k

**Published:** 2020-12-24

**Authors:** Karoline de Sousa Castro, Luís Fernando de Medeiros Costa, Valter José Fernandes, Regineide de Oliveira Lima, Aruzza Mabel de Morais Araújo, Mikele Cândida Sousa de Sant'Anna, Nataly Albuquerque dos Santos, Amanda Duarte Gondim

**Affiliations:** Federal University of Rio Grande do Norte, Sciences and Petroleum Engineering Graduate Program Natal RN 59078-970 Brazil karol-castro76@hotmail.com; Federal University of Rio Grande do Norte, Institute of Chemistry Natal RN 59078-970 Brazil amandagondim.ufrn@gmail.com; Federal University of Maranhão, Department of Fishing Engineering Pinheiro MA 65200-000 Brazil mikelecandida@gmail.com; Federal University of Paraíba, Department of Food Technology João Pessoa PB 58.051-900 Brazil natalyjp@gmail.com

## Abstract

The present work aims to evaluate the potential of Al-MCM-41 and Ni/Al-MCM-41 catalysts for the production of renewable hydrocarbons through the fast pyrolysis of palm oil. Al-MCM-41 mesoporous material was synthesized by the hydrothermal route. The Ni/Al-MCM-41 catalyst was obtained by the wet impregnation method of the Al-MCM-41 material (support) previously synthesized with 2.3% metal in relation to the support mass. The thermal pyrolysis of palm oil yielded many oxygenated compounds with a very high molecular mass. The pyrolysis of the oil under the action of Al-MCM-41 presented greater selectivity when compared to thermal pyrolysis, obtaining 63% of hydrocarbons in the C11–C15 region. The catalytic pyrolysis of the oil with Ni/Al-MCM-41 showed a high deoxygenation rate, obtaining a hydrocarbon percentage equal to 78%, in addition to obtaining a percentage of hydrocarbons equal to 46% in the region of interest, *viz.*, C11–C15, demonstrating the potential of the Ni/Al-MCM-41 catalyst for renewable hydrocarbons production (bio-jet fuel) from palm oil.

## Abstract 2

The potential of the Al-MCM-41 and Ni/Al-MCM-41 catalysts applied to pyrolysis for the production of renewable hydrocarbons (bio-jet fuel) has been studied and has been shown to be efficient.

## Graphical abstract

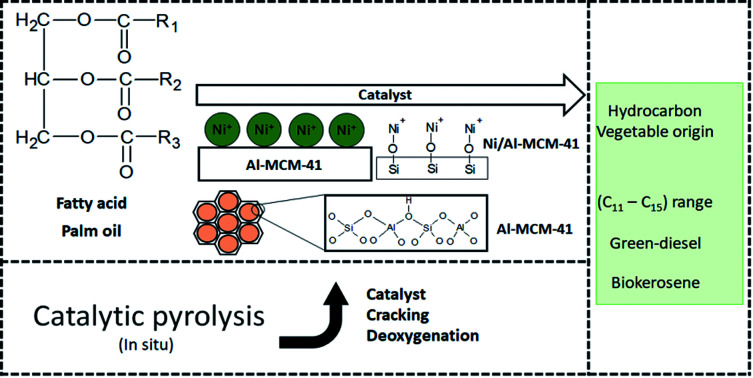

## 1. Introduction

Global aviation consumes about 220 Mt y^−1^ of fuel, accounting to a contribution of 2.6% of anthropogenic CO_2_ emission, which could potentially reach 4.6% by 2050, according to the updated data from the Intergovernmental Panel on Climate Change (IPCC).^1–3^ Also, jet fuels or kerosene are classified as the largest emitter of greenhouse gases per transport unit.^4^ These facts, together with the very limited options available to make aviation greener, make the goal of developing sustainable biofuels attractive; however, technical issues and economic competitiveness continue to pose real challenges.^2^

Unlike the application of alternative fuels in other sectors, jet fuels have a much greater constraint in their substitution. First, the extreme conditions under which the fuel works require it to be within specifications, restricting alternative fuel options. Secondly, to avoid logistic problems and commercial limitations at airports with fuels of different grades, any proposed product should be fully replaceable by the current aviation fuel. Finally, the long service life of the commercial aircraft means that any candidate fuel must be retrofitted and suitable for use in engines with the existing technology. For these reasons, the main focus of research has been to develop drop-in fuels, which can be used in the existing fleet.^5^

Currently, five methods are certified by the American Society for Testing Materials (ASTM) for the production of aviation biofuels in ASTM D7566.^6^ Today, the certified methods for obtaining bio-jet fuel are synthesized paraffinic kerosene by Fischer–Tropsch (FT-SPK), hydroprocessed esters and fatty acids (HEFAs), synthesized iso-paraffinic (SIP), synthesized paraffinic kerosene by Fischer–Tropsch with aromatic (FT-SPK/A), alcohol-to-jet (ATJ), and catalytic hydrothermolysis jet (CHJ). Pyrolysis catalytic and hydrogenated pyrolysis oil (HPO) are a possibility; however, studies are still needed so that the route can be certified.^7^

Catalytic pyrolysis oil (CPO) or hydrogenated pyrolysis oil (HPO), containing hydrogen, can be effectively applied to lipids and fatty acids to remove oxygen and generate hydrocarbons.^8^ The main advantage is the possibility of using low-cost catalysts and/or does not require hydrogen. HPO and CPO processes are based on deoxygenation reactions (decarbonylation, decarboxylation, hydrodeoxygenation, cracking, hydrocracking, and among others) to convert fatty acids into hydrocarbons. However, in literature, the CPO process has encountered the problem of excessive deactivation of the catalyst by coke formation, mainly with zeolites (microporous).^9^ Several catalysts have been studied to produce hydrocarbons from oils and fats. Among the main catalysts are zeolites,^10^ metal oxides,^11^ pillared clays,^12^ silica,^13^ alumina,^14^ and the mixture of these.

In 2012, Wang and collaborators^15^ studied two supports (Al_2_O_3_ and mesoporous zeolite) for Pt with a catalyst for CPO and hydrodeoxygenation of the oil (HDO), and a higher degree of deoxygenation was observed for the mesoporous material. Other authors have studied mesoporous materials that have been used as heterogeneous catalysts to obtain biofuels.^2,16^

MCM-41 (ref. 17) is one of the most recent members of the mesoporous family of materials and has been widely used^16,18,19^ as it has a high surface area due to the highly ordered one-dimensional hexagonal structure proposed by Beck and collaborators.^20^ The catalyst has a uniform hexagonal matrix of mesopores, high surface area, and moderate acidity. Because of these properties, MCM-41 materials are currently under study in a variety of processes as catalysts or as catalyst carriers.

The presence of the MCM-41 material significantly alters the quality of the pyrolysis products, while Al-MCM-41 decreases the fraction of undesirable oxygenated compounds in the bio-oil produced.^17^ Besides, the thermal stability of this material makes it feasible for various applications. The incorporation of noble metals (Pd, Pt, Ru, Ir, Os, and Rh) and non-noble metals (Ni) in the material promote the deoxygenation catalytic of triglycerides.^21^

The most common metals are aluminium and nickel, which favor chain break reactions. Combined with the increase in the temperature of the pyrolysis process, they together constitute the process of thermo-catalytic cracking.

Among the several biomasses used for the production of biofuels, palm oil has a great energy potential, with the content of oil extracted in the range from 45 to 55 wt%.^22^ The fatty acid with the highest concentration in palm oil is palmitic acid (C16:0), with about 44% concentration by mass.^22^ Thus, it is possible to use it as a raw material for renewable hydrocarbons to produce bio-jet fuel since its composition, after the deoxygenation processes, will be within the bio-jet fuel range, with respect to the size of the carbon chain. In this work, palm oil was used in the production of bio-jet fuel (drop-in fuel) through the catalytic pyrolysis of the oil (CPO) from Ni/Al-MCM-41 and Al-MCM-41 catalysts.

## 2. Experimental

### 2.1. Al-MCM-41 and Ni/Al-MCM-41 synthesis

The catalysts were synthesized from the following reagents: silica gel (SiO_2_, 95%, Aldrich), cetyltrimethylammonium bromide (CTMABr, 98%, Vetec), sodium hydroxide (NaOH, 99%, Vetec), aluminium oxide (Al_2_O_3_, 99,9%, Aldrich), and acetic acid (CH_3_COOH, 99,7%, Merck) according to Schwanke *et al.*^23^ The reactants were added in stoichiometric proportions and pre-defined orders to produce a gel having the following molar composition: 1CTMABr : 2NaOH : 4SiO_2_ : 200H_2_O : 0.04Al_2_O_3_. For the production of 200 g gel, two solutions were prepared: (i) 11.30 g of silica, 3.76 g of sodium hydroxide, 0.19 g of aluminium oxide, and 84.6 g of water were placed in a 250 mL beaker and stirred for 2 h in the temperature range of 60–70 °C; (ii) 17.13 g of cetyltrimethylammonium bromide and 84.6 g of water were placed in another 250 mL beaker and stirred for 30 min at room temperature. Solution (ii) was added to solution (i) and stirred for 30 min at room temperature. The new blend was placed in a Teflon autoclave for 48 h at 100 °C, pH (9.0–10.0) corrections being made daily with the acetic acid solution. The material was then filtered, washed with distilled water, and dried at 100 °C for 4 h.

Employing thermogravimetric analysis (TGA/DTG) of the Al-MCM-41 material, the calcination curve was determined, which was aimed at the removal of the CTMA^+^ ions from the organic driver. The non-calcined material was used to obtain the TGA/DTG curves, in which it was heated to 900 °C at a heating rate of 10 °C min^−1^ under a helium flow of 25 mL min^−1^ in alumina crucibles with approximately 20 mg of sample. The TGA/DTG curves obtained were evaluated.

Using the TGA/DTG data, Al-MCM-41 was calcined at 450 °C with a heating ramp of 5 °C min^−1^ for 1 h under nitrogen atmosphere; then, the atmosphere was changed to synthetic air and maintained for a further 1 h at the same temperature.

To obtain Ni/Al-MCM-41, with 9 wt% of the metal in relation to the support, the materials used were Al-MCM-41, nickel nitrate hexahydrate (NiNO_3_·6H_2_O, 97%, Merck), and ethanol (CH_3_CH_2_OH, 99.7%, Dinâmica). Nickel nitrate was dissolved in 0.05 L ethanol at a concentration of 3.49 g L^−1^, placed in an Erlenmeyer flask, Al-MCM-41 was added thereto, which was maintained under stirring for 2 h at room temperature and evaporated at 80 °C. The material was filtered, washed with distilled water, and dried at 60 °C. Al-MCM-41, after impregnation of Ni with excess solvent, was heated in a muffle furnace to a temperature of 550 °C at a heating rate of 1 °C min^−1^ and maintained for four hours under a nitrogen flow of 75 mL min^−1^.^24^

### 2.2. Characterization of the catalysts

The catalysts were characterized by X-ray diffraction (XRD), thermogravimetric analysis (TGA/DTG), Fourier transform infrared spectroscopy (FTIR), and scanning electron microscopy coupled to energy dispersive spectroscopy (SEM/EDS).

The X-ray diffractograms of the materials were obtained through the model D2 PHASER BRUKER equipment, with a CuKα radiation source, a 30 kV voltage nickel filter, 30 mA tube current, and Lynxeye detector, using the powder method in the 2*θ* range of 1–10° with a scan rate of 2° min^−1^ and a pitch of 0.01°.

The thermogravimetric analysis (TGA/DTG) for these samples was performed using a model 851e Mettler Toledo TGA/SDTA thromboxane. The TG curves were obtained using alumina crucibles in the temperature range from ambient up to 900 °C, using a heating rate of 10 °C min^−1^ and under a nitrogen atmosphere (25 mL min^−1^).

The infrared absorption analyses were performed using a SHIMADZU equipment, model IRAFFINITY, with potassium bromide (KBr) as the dispersing agent in the range of 4000–400 cm^−1^.

The scanning electron microscopy (SEM) images of the catalysts were obtained from a HITACHI apparatus, model TM-3000, where the materials were placed in a sample holder on a carbon tape. Analyses were performed using 500 to 8000-fold magnification with 2 to 4-fold digital zoom. Coupled to SEM, the energy dispersive spectroscopic (EDS) analyses of mesoporous materials were performed using an OXFORD SWIFTED 3000 model, aiming to provide semi-quantitative information of the elements present in the samples.

### 2.3. Characterization of palm oil

Palm oil was purchased from a commercial establishment and was produced by ICPA CEPERA LTDA. The characterization of the oil was performed for the parameters of acidity index, lipid composition by gas chromatography, oxidative stability (RANCIMAT and PDSC), and Fourier-transform infrared spectroscopy (FTIR).

To perform the acidity index test, 2 g of oil was weighed and dissolved in 25 mL of an ethyl ether/ethanol solution (2 : 1), adding 3 drops of 1% phenolphthalein indicator and 0.1 M NaOH as the titrant; the test was performed in triplicate.^25^

For the determination of the fatty acid profile, methyl esterification was performed following the methodology described by Hartman and Lago.^26^ The quantification was obtained by calibration with standards of fatty acid methyl esters (Supelco® 37 Component FAME Mix), using gas chromatography (GCMS-QP2010 Shimadzu, Kyoto, Japan) equipped with a Durabound DB-23 (30 m × 0.25 mm × 0.25 μm) column. All the tests were performed in triplicate.

The oxidation temperature (OT) was measured using a differential scanning calorimeter pressure (PDSC) (NETZSCH, MDSC model 2920) through a dynamic DSC curve, using a heating rate of 10 °C min^−1^ from room temperature to 470 °C, pressure 1100 kPa, in an atmosphere of oxygen.

The oxidative stability was studied using a METROHM equipment, model Rancimat 843, in which the oil was maintained at a constant temperature of 110 °C and the air flow of 10 L h^−1^.

The Fourier transform infrared (FTIR) spectra were obtained using the Attenuated Total Reflectance (ATR) technique using the 45° ZnSe cell in the transmittance mode in the range of 4000 to 700 cm^−1^ with the resolution of 4 cm^−1^ employing a Bomem Fourier transform infrared spectrophotometer, model MB102. The blank test was performed using the ZnSe cell with a sample and to obtain the ATR/FTIR spectra, a volume sufficient to cover the cell was used.

### 2.4. Catalytic pyrolysis process

Palm oil was subjected to thermal pyrolysis and thermo-catalytic pyrolysis in a model PY-2020Is Control SHIMADZU pyrolyzer, which was connected online to a gas chromatograph with mass spectroscopy, model QP 2010 (Frontier Lab). Pyrolysis occurred at 500 °C for 1 min; the products were separated by chromatography and identified by a mass spectrometer. The samples were placed in a stainless-steel crucible (pyrolyzer) and under a helium atmosphere with 3 mL min^−1^ flow, the analysis was performed by the split mode in the 200 : 1 ratio and the injector temperature of 250 °C. An RTX-1 PONA (100% dimethylpolysiloxane) column was used with a length of 100 m, a diameter of 0.25 mm, and a stationary phase width of 0.5 μm. The pressure of the column used was 116.7 kPa with a flow rate of 2.20 mL min^−1^ and a linear velocity of 53.2 cm s^−1^. The chromatographic oven was programmed (temperature 30 °C for 5 min up to the temperature of 320 °C) and the heating rate was 10 °C min^−1^, and the schedule was 46 min. The GC/MS interface temperature was 250 °C and the mass spectrometer detection was between 20 and 600 *m*/*z*, while the scan interval was 0.50 s and the speed was 1250 μm s^−1^. This Py-GC/MS methodology was reported by Araújo and collaborators.^27^ For thermocatalytic pyrolysis, 10% by mass of the catalyst was added to the palm oil and mixed physically until homogenization. Furthermore, through this analysis, the percentage in terms of the peak area (% peak area) of each constituent was calculated. The total sum of *n* (∑*n*), where *n* equal to the percentage of peak area of each constituent in the sample, is equal to 100%.

## 3. Results and discussion

### 3.1. Characterization of the catalysts

**Fig. 1:**
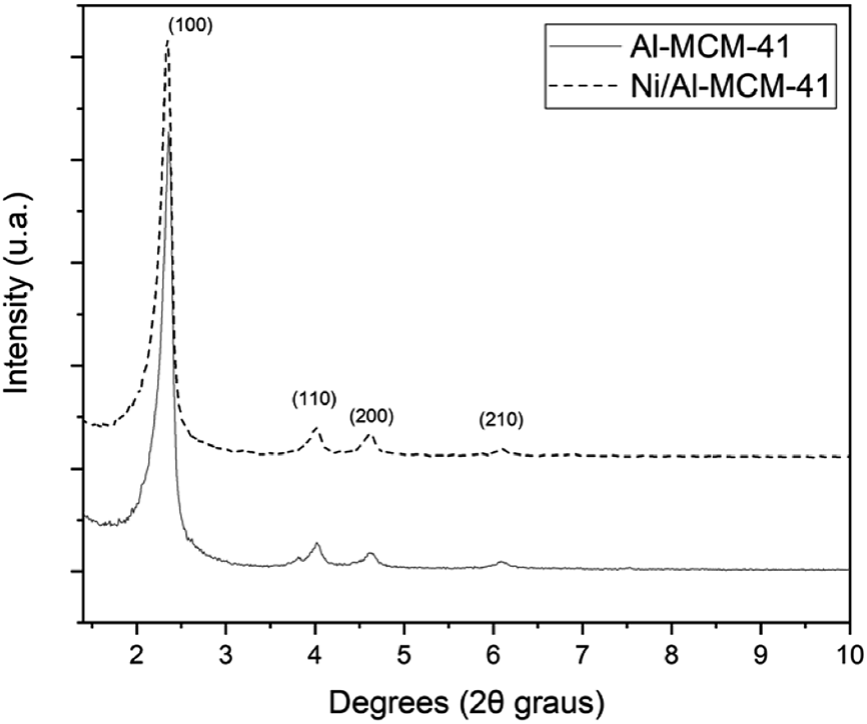
X-ray diffractograms of the Al-MCM-41 and Ni/Al-MCM-41 catalysts.

The XRD patterns of the catalysts Al-MCM-41 and Ni/Al-MCM-41 (Fig. 1) presented four characteristic peaks for the structure of mesoporous type of MCM-41. The strongest peak is in the range of 2*θ* = 2.5° due to the *d*_100_ plane.^28^ Besides, small peaks due to reflections from higher-order planes (110), (200), and (210) indicated the formation of a mesoporous material with three signals in the range of 4 and 6.5°. These reflections characterize the formation of the one-dimensional hexagonal structure proposed by Beck and collaborators.^20^ The introduction of nickel in the catalyst structure was noted by the slight decrease in the in-plane intensity (100) about the other catalyst, showing a slight decrease in the hexagonal symmetry due to a greater disturbance in the structure, which is attributed to the inductive effect of the metal on the structure, as found by Fu and collaborators.^29^

**Fig. 2:**
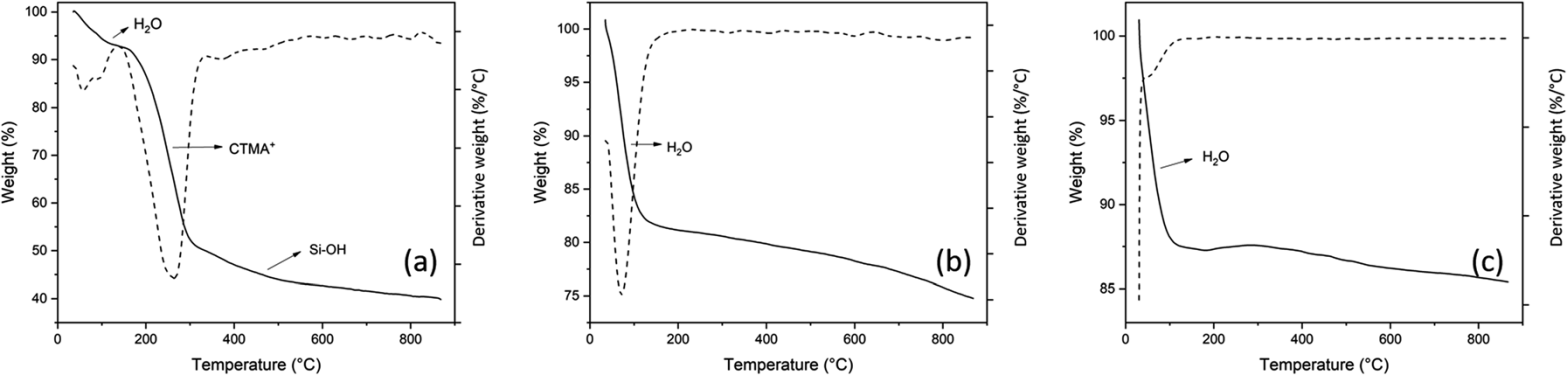
The TGA/DTG curves for non-calcined (a) calcined (b) AlMCM-41 and (c) Ni/Al-MCM-41.

The TGA/DTG curves of the Al-MCM-41 sample before the calcination process, after the calcination process, and Al-MCM-41 impregnated with the calcined nickel metal are shown in Fig. 2. For all these samples, the first loss of mass is attributed to the desorption of water absorbed on the external and internal surface of the material structure in the temperature range from 37 °C to 150 °C, with mass losses of approximately 7 to 12% for the samples of non-calcined Al-MCM-41 and Ni/Al-MCM-41. Regarding the calcined Al-MCM-41 sample, a percentage close to 18% of the mass loss was observed; this difference is attributed to the exposure of the sample to moisture before the thermogravimetric analysis since the mesoporous silica-based material is susceptible to physically adsorbed water.^30^ The second decay in the temperature range between 139 °C and 415 °C, comprised the decomposition of the organic driver (CTMABr), comprises the largest loss in the mass of the sample of approximately 42%. The third loss in the mass was about 9% in the range from 415 °C to 800 °C, which was determined only by the derivative of the thermogravimetric curve and is related to the decomposition of the surfactant residues.^30^ For the calcined Al-MCM-41 sample, no loss in the mass corresponding to the decomposition of the organic driver was observed, indicating that the calcination process was effective in removing the surfactant. In addition, a slow loss of mass from 114 °C was noted, which may correspond to the release of water absorbed by the material.^31^ The thermal stability of the Ni/Al-MCM-41 sample is observed in Fig. 2(c), where a residual mass of 85% is identified with approximately 12% of the mass loss in the range of 37–120 °C, which is attributed to the absorbed water by the catalyst. Also, only a loss of approximately 3% by mass was used to decompose the Ni/Al-MCM-41 produced, as found by Franco and collaborators.^32^

**Fig. 3:**
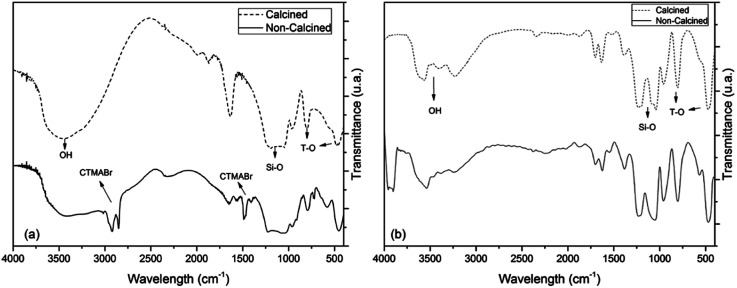
TGA/DTG curves for non-calcined (a) AlMCM-41 (b) Ni/AlMCM-41.

The FTIR of the catalysts was analyzed before and after the calcination process; the data after the nickel-metal impregnation in the calcined Al-MCM-41 data is shown in Table 1 and Fig. 3. The spectrum of Al-MCM-41 before the calcination process shows bands in the range of 3000–2800 cm^−1^ and 1500–1445 cm^−1^, which correspond to the stretching and reflection vibrations of the C–H bonds of the CH_2_ and the CH_3_ groups of the aliphatic chain of the structure template, respectively. On the other hand, these bands were absent in the calcined sample, implying that the calcination process effectively removed the template organic as found by La-Salvia and collaborators.^33^ Continuing in the analysis of the FTIR spectrum of the calcined Al-MCM-41 catalyst, bands between 3690 and 3200 cm^−1^ and the band at 1644 cm^−1^ is attributed to the stretching of the O–H bonds of the hydroxyl groups on the surface of the material and due to the adsorbed water molecules.^34^ The bands in the region between 1211 and 1032 cm^−1^ and the bands at 797 and 470 cm^−1^ correspond to the asymmetric and symmetrical stretching vibrations, respectively, of the T–O–T (T = Si or Al) connections.^35^ An indication of the presence of aluminium in the mesoporous structure is related to the presence of the 951 cm^−1^ bands.^36^ Regarding the FTIR spectrum of the Ni/Al-MCM-41 catalyst, no bands were observed directly for nickel; however, there is the presence of the 1397 cm^−1^ band caused by the presence of NO_3_, which originates from the use of nickel nitrate, which indicates the presence of nickel in the support.^37^ The summary of the FTIR data is shown in Table 1.

**Table 1:** Assignments of the Al-MCM-41 and Ni/Al-MCM-41 infrared bands

Sample	Bands	Assignments
Al-MCM-41 before calcination	3000–2800 cm^−1^	Stretching vibrations of the C–H groups of the CTMA^+^ species
	1500–1445 cm^−1^	Reflection vibrations of the C–H of the groups CTMA^+^ species
Al-MCM-41 after calcination	3690, 3200, 1644 cm^−1^	Stretching vibrations of O–H
	1211 cm^−1^ and 1032 cm^−1^	Asymmetric deformation of T–O–T*
	797 cm^−1^ and 470 cm^−1^	Symmetric deformation of T–O–T*
	951 cm^−1^	Presence of Al in the structure
Ni/Al-MCM-41	1397 cm^−1^	Presence of NO_3_

**Fig. 4:**
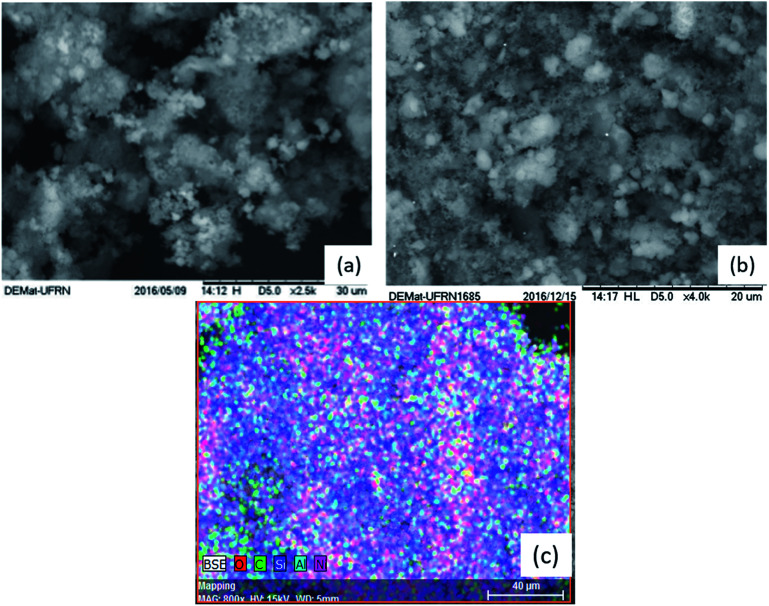
Scanning electron micrograph of Al-MCM-41 (a) and Ni/Al-MCM-41 (b); EDS analysis of Ni/Al-MCM-41 (c).

The morphology of the Al-MCM-41 samples after the calcination process and the Ni/Al-MCM-41 calcined samples is shown in Fig. 4. In Fig. 4(a), some agglomerated particles with irregular shapes are observed, as well as spherical shapes with a homogeneous appearance, which is considered as evidence of the incorporation of aluminium in the structure of MCM-41.^38,39^Fig. 4(b) shows the increase in the particles agglomerated on the surface of the material, which is an indication of the presence of nickel metal particles.^40^Fig. 4(c) shows the picture obtained from the EDS analysis for the calcined Ni/Al-MCM-41 sample. The analysis confirmed the presence of the elements expected for all the materials, mainly the presence of nickel metal in the Ni/Al-MCM-41 sample, presenting 2.3% of this metal in the analyzed region. This value is in disagreement with the percentage value of the impregnated metal in this study; however, this may be related to the inhomogeneity of the metal in the chosen area of analysis or the loss of matter during the impregnation process, as found by Rahmanzadeh and Taghizadeh.^40^

### 3.2. Characterization of palm oil

The acid value of palm oil was 0.70 mg NaOH per g, indicating the low degradation of the glycerides present. This value is close to the value found in the study by Wang *et al.*,^41^ in which the acid value of 1.14 mg NaOH per g for palm oil was verified. The proportion of fatty acids in palm oil is described in Table 2. Given these values, it is observed that the main acids identified were linoleic, followed by palmitic. These values were compared with the data from Mancini and collaborators^42^ as well as Aung and collaborators.^43^ A discrepancy in the data was observed regarding palmitic acid, which is present in a higher concentration in the oil used in the study by Mancini and collaborators,^42^ followed by oleic acid. However, Aung and collaborators^43^ showed a higher concentration of oleic acid, followed by palmitic acid.

**Table 2:** Fatty acid composition of palm oil

Components	Fatty acids of palm oil (%)		
	Sample (this work)	Mancini *et al.* (2015)	Aung *et al.* (2018)
Lauric acid (C12:0)	1.6	0.2	0.2
Myristic acid (C14:0)	2.9	1.1	0.7
Palmitic acid (C16:0)	27.3	44	27.1
Palmitoleic acid (C16:1)	—	—	0.1
Stearic acid (C18:0)	6.5	4.5	2.9
Oleic acid (C18:1n9t)	2.5	39.2	32.8
Linoleic acid (C18:1n9c)	46.4	10.1	15.1
Linolenic acid (C18:2n6c)	12.8	0.4	0.2

**Fig. 5:**
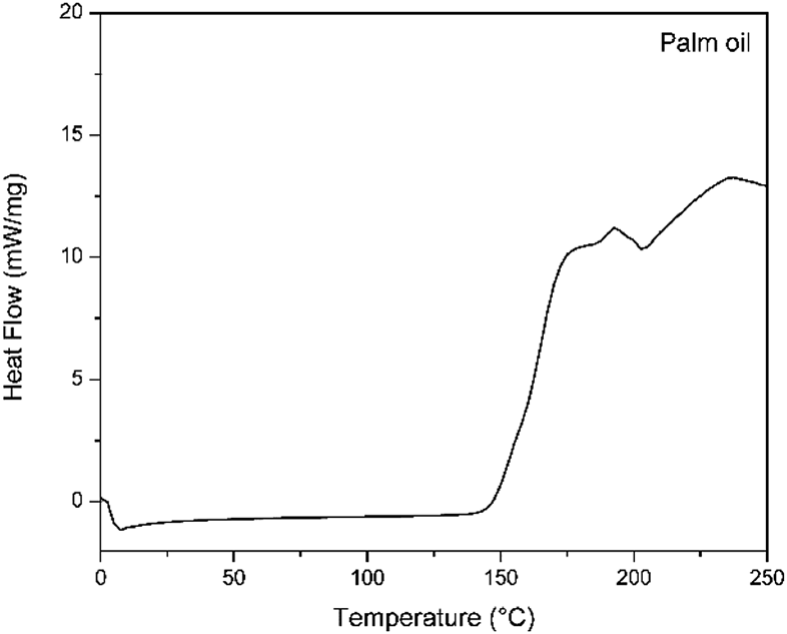
PDSC curve of palm oil.

The onset temperature of palm oil was about 150 °C, which we call the oxidation temperature (OT). Fig. 5 shows the dynamic PDSC curves. The onset of oxidation is evidenced by the first sharp increase in the value of the heat flow, which occurs at about 150 °C. The high-intensity peak indicates the phenomenon of combustion, in which all the oil is degraded. Galvão and collaborators^44^ determined the OT value of sunflower and cotton oil, which was to be 168.2 and 166.4 °C, respectively. The oxidative stability depends on the chemical composition, the quality of the raw material, and the conditions of the refining processes, and the OT value can be used for evaluating the quality of oils and fats.^44^

**Fig. 6:**
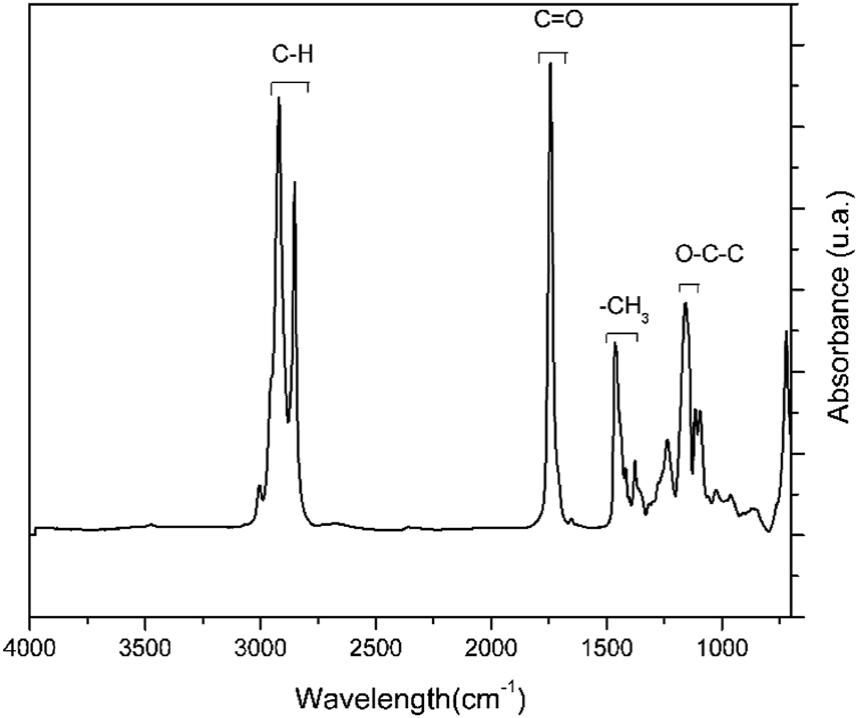
The FTIR spectrum of palm oil.

The FTIR spectrum showing the characteristic bands of the lipid chain of palm oil is shown in Fig. 6 and Table 3. The low-intensity band at 3007 cm^−1^ is attributed to the vibration of the asymmetric C–H bonds of the carbon–carbon double bond. The two intense bands at 2929 cm^−1^ and 2856 cm^−1^ are related to the asymmetric and symmetrical stretching vibration, respectively, of the C–H aliphatic bonds.^45^ The band of greater intensity at 1746 cm^−1^ corresponds to the stretching vibrations of the carbonyl functional group (C=O), while the bands at 1462 cm^−1^ and 1378 cm^−1^ are related to the asymmetric and symmetrical vibrations of the CH bonds of the CH_3_ groups, respectively.^45^ Another intermediate band at 1161 cm^−1^ refers to the asymmetric axial stretching vibrations of the O–C–C bonds of the ester groups.^46^ Finally, the band at 726 cm^−1^ is attributed to the planar flexion of the C–H connections.^47^ The FTIR spectrum of palm oil displayed three main bands (Fig. 6).

**Table 3:** Assignments of the infrared bands of palm oil

Bands	Assignments
3007 cm^−1^	Asymmetric vibrations of the C–H bond of –CH=CH–
2929 cm^−1^	Asymmetric stretching vibration of the C–H bond
2856 cm^−1^	Symmetric stretching vibration of the C–H bond
1746 cm^−1^	Stretching vibration of the C=O bond
1462 cm^−1^	Asymmetric vibration of the C–H bond of the CH_3_ groups
1378 cm^−1^	Symmetric vibration of the C–H bond of CH_3_ groups
1161 cm^−1^	Axial vibration of the asymmetric stretching of the O–C–C bond
726 cm^−1^	Planar flexion of the C–H bond

The oil oxidative stability by Rancimat test was performed in duplicate; the mean value obtained was 6 h 12 min for the beginning of degradation at a temperature of 110 °C, thus presenting excellent stability. Yagcı and collaborators^48^ performed the Rancimat test at 90, 100, and 120 °C and obtained an induction period of 611, 457, and 254 min, respectively. The value of 254 min (4.24 h) obtained at 120 °C is lower than that determined in this work, which is consistent.

### 3.3. Catalytic pyrolysis processes

**Fig. 7:**
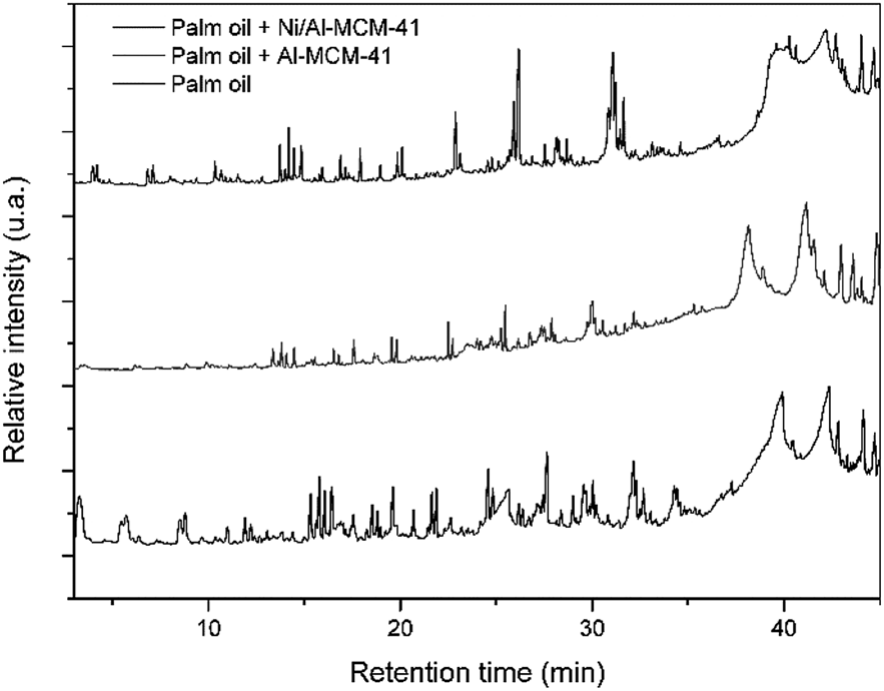
The pyrograms of the products captured in the thermal and thermo-catalytic pyrolysis of palm oil on the two studied catalysts (Al-MCM-41 and Ni/Al-MCM-41).

The oil was subjected to thermal pyrolysis and catalytic pyrolysis processes under the action of previously synthesized Al-MCM-41 and Ni/Al-MCM-41 catalysts. The pyrograms of the components of each pyrolysis process is shown in Fig. 7.

The Py-GC/MS data indicate a difference in the composition of the products obtained in the thermal and catalytic cracking, contradicting the idea that the presence of catalysts does not significantly alter the composition of the pyrolysis products of vegetable oil, as reported in the literature.^2,18^ On the other hand, it demonstrates that the distribution of products is modified by the action of the catalysts.

The intensity of the peak referenced to the products with higher molar mass (longer retention time) in the pyrogram derived from the thermo-catalytic process under the action of Ni/Al-MCM-41 is relatively higher when compared to the same peaks in the pyrogram from the thermal process. On the other hand, the peak intensity for Al-MCM-41 was less intense when compared to that for Ni/Al-MCM-41. This is probably attributed to the selectivity of the products of this catalyst.

Mesoporous materials due to their pores with sizes between 2 and 50 nm are known for their ability to crack down high molecular weight compounds.^49^ This led to the development of catalysts with large pores, which facilitates the entry of larger molecules, such as triglycerides; however, these suffer the consequence of their low acid nature, which is not very suitable for pyrolysis reactions.^50^ Through the impregnation of metals such as Al and Ni in action with the mesoporous reorganization of MCM-41, we seek to supply the need for the catalytic activity around the acidity and selectivity of products obtained through the catalytic pyrolysis reaction.^19^

Among the products obtained, the presence of organic acids, alcohols, aldehydes, and esters and ketones can be observed, besides alkanes and alkenes in the hydrocarbon range, as found by Ma and collaborators.^51^ Most chemical substances are as follows: 2 propen-1-ol, 1-heptene, ethyl-cyclohexane, nonane, butanic acid, 1,3-octadiene, undecane, 4-methyl-1-undecane, 2-propenyl ester-octanoic acid, heptadecane, heneicosane, and hexacontane.

In the products formed, it was observed that a majority of hydrocarbons and a percentage of oxygenates decrease with the use of Ni/Al-MCM-41 (thermo-catalytic). As can be seen in Table 4, the pyrolysis catalyzed in the presence of Ni/Al-MCM-41 showed a better response to the reduction of oxygenates. This result was attributed to the active acid sites caused by the impregnation of Ni, which was efficient for the deoxygenation of the oil under pre-established conditions.^51^ The impregnation of metals allows the generation of charges on the surface of the mesoporous material and consequently improves the acidity as discussed through the microcalori-metric NH_3_ adsorption studies performed by Kumaran *et al.*^57^ Therefore, the impregnation of Ni in Al-MCM-41 provides interaction between the catalyst and the oil, consequently improving the activity in the deoxygenation process. The deoxygenation catalytic process for the sample catalyzed by Al-MCM-41 was not so satisfactory due to the lower acidity of the catalyst.^2,18,52^ The increase in aluminium in the structure is responsible for the Lewis sites and is characterized by the charge deficiency generated by the presence of an Al atom isomorphically substituted in the place of an Si atom. The Brönsted sites are attributed to the H^+^ ion present in the structure; however, the insertion of aluminum in the MCM-41 structure did not promote sufficient acidic sites for an effective deoxygenation process.

**Table 4:** Percentage of hydrocarbons and oxygenates of the pyrolysis products

Samples	Hydrocarbons (%)	Oxygenated compounds (%)
Pure oil	64.5	35.5
Oil + Al-MCM-41	61.0	39.0
Oil + Ni/Al-MCM-41	78.0	22.0

Table 5 shows that the selectivity of hydrocarbons is related to the use of catalysts with satisfactory pore diameter and active acid sites.^53^ When analyzing the thermal process, it can be seen that there is no selectivity between the analyzed ranges and a percentage of 34.5% of the products with a chain above 15 carbons, which is not satisfactory for use as biofuels.

**Table 5:** Percentage of hydrocarbon fractions of the pyrolysis products

Samples	C3–C5 (%)	C6–C10 (%)	C11–C15 (%)	>C15 (%)
Pure oil	3.4	27.6	34.5	34.5
Oil + Al-MCM-41	0.0	25.2	63.0	11.8
Oil + Ni/Al-MCM-41	2.7	28.2	46.0	23.1

For the catalytic processes, there was a selectivity on the part of the catalysts; in the test carried out by the pyrolysis catalyzed with Al-MCM-41, it presented a satisfactory percentage of products with added values for the petrochemical industry, with a higher percentage in the fraction of aviation biokerosene (C11–C15). The sample catalyzed by Ni/Al-MCM-41 also presented a higher percentage of hydrocarbons in the aviation biokerosene range but with a notable percentage of 23% of hydrocarbons in the range above C10, which is a range classified as green gasoline.

Another factor that greatly influences the composition of the products is the composition of the raw material, which is, in this case, vegetable oil. Oils and fats with a high amount of unsaturated fatty acids and relatively small carbon chains favor obtaining the gasoline fraction with a high content of aromatics.^52^ Unsaturation facilitates the cracking of the carbon chain and the reactions of cyclization and later aromatization.^53^ Those with a high content of saturated fatty acids and with large carbon chains favor obtaining the green-diesel fraction or aviation biokerosene with fewer aromatics.^54^

**Fig. 8:**
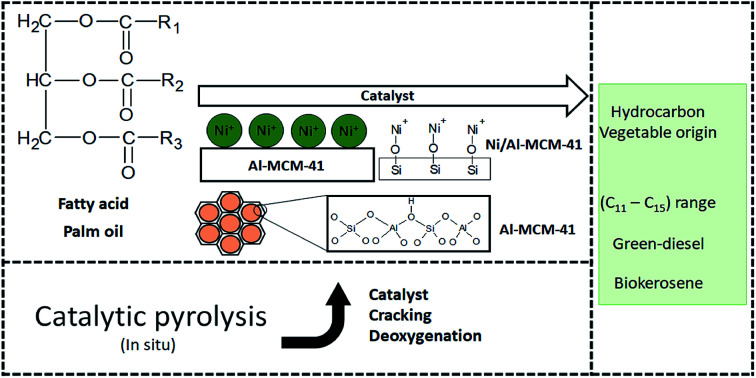
A summary of the pyrolysis mechanism with Al-MCM-41, Ni/Al-MCM-41, and palm oil.

In the mechanism regarding the decomposition of palm oil by catalytic pyrolysis, triglycerides and fatty acids can form a variety of oxygenated hydrocarbons and unsaturated hydrocarbons. In this process, it has the possibility to present selectivity in the reaction paths and certain compounds or hydrocarbon ranges. In addition, hydrocarbons continue to decompose to form others hydrocarbons of smaller size. The hydrocarbons are oxygenated by decarboxylation, decarbonylation, and β-scission, and unsaturated hydrocarbons are converted into other small molecule hydrocarbons by β-scission and elimination, as discussed by Qiao *et al.*^55^ Thus, different hydrocarbons can be converted by different types of isomerization, polymerization, and cyclization reactions. Thus, AlMCM-41 and Ni/Al-MCM41 enabled the formation of compounds in the hydrocarbon range of biokerosene and green-diesel detected in the PY/GC-MS analysis. Fig. 8 shows a summary of the pyrolysis mechanism with Al-MCM-41, Ni/Al-MCM-41, and palm oil.

In summary, mesoporous materials have an amorphous characteristic, as evidenced by XRD; in addition, it is known that these purely silicic materials have a low acidity, as stated by Meziani *et al.*^56^ Calcined aluminosilicates, when subjected to TGA/DTG, present both Lewis and Brönsted acid sites, as discussed by Corma *et al.*^57^ The increase in aluminum in the structure is responsible for the Lewis sites and is characterized by the charge deficiency generated by the presence of an Al atom isomorphically substituted in the place of an Si atom. Brönsted sites are attributed to the H^+^ ion present in the structure. As observed in the FTIR spectrum, the T–O–T bond (T = Si or Al) is an important interaction to promote the decomposition of fatty acid molecules caused by the active sites, which leads to the selectivity of Al-MCM-41. The formation of carbocations by various reaction steps happens to break due to the effects of the temperature and catalyst. Thus, the impregnation of metals allows the generation of charges on the surface of the mesoporous material and consequently improves the acidity, as discussed in Kumaran *et al.*^58^ Therefore, the impregnation of Ni in Al-MCM-41 provides interaction between the catalyst and the oil, consequently improving the activity in the deoxygenation process.

## 4. Conclusions

The synthesis of Al-MCM-41 with an Si/Al ratio of 50 using the hydrotreatment method proved to be viable since the mesoporous material obtained showed properties similar to those exposed in the studies. The impregnation process employs nickel nitrate hexahydrate by the wet method. The results from several characterization techniques such as thermal analysis, X-ray diffraction, scanning electron microscopy, and Fourier transform infrared spectroscopy indicate the obtaining of the mesoporous material Al-MCM-41 with organized amorphous structure with the arrangement of one-dimensional hexagonal defined, showing the diffraction planes of (100), (110), (200), and (210). Thermogravimetric analysis confirms that at temperatures above 415 °C, the organic template, CTMABr, is eliminated from the Al-MCM-41 sample. Consequently, when the support is subjected to calcination at 450 °C for 1 h, the resulting material is free of the surfactant, which is proven in the infrared spectrum, while dispersive energy spectroscopy indicated the presence of metals such as aluminium and nickel in the respective catalysts. Palm oil, according to the physicochemical characteristics, is suitable for the conversion process. Thermal oxidative stability demonstrated satisfactory values as well as the acidity index, indicating a low degradation of its components. The Rancimat test indicated that the oil can be subjected to long storage times at room temperature. Catalytic pyrolysis with Al-MCM-41 and Ni/Al-MCM-41 catalysts presented better results than thermal pyrolysis, both for the formation of hydrocarbons and for the production of compounds in the C11–C15 (63% and 46%, respectively) region of interest. The Al-MCM-41 catalyst showed high selectivity in the region of interest, while the Ni/Al-MCM-41 catalyst demonstrated efficiency in the deoxygenation process of fatty acids, which obtained the highest percentage of hydrocarbons (78%). Therefore, the Ni/Al-MCM-41 catalyst shows good results for renewable hydrocarbon production (bio-jet fuel) from palm oil.

## Conflicts of interest

There are no conflicts to declare.

## Supplementary Material
